# Awareness and knowledge among internal medicine house-staff for dose adjustment of commonly used medications in patients with CKD

**DOI:** 10.1186/s12882-017-0443-7

**Published:** 2017-01-17

**Authors:** Sikander Surana, Neeru Kumar, Amita Vasudeva, Gulvahid Shaikh, Kenar D. Jhaveri, Hitesh Shah, Deepa Malieckal, Joshua Fogel, Gurwinder Sidhu, Sofia Rubinstein

**Affiliations:** 1 Nephrology Division, Department of Medicine, Nassau University Medical Center, 2201 Hempstead Turnpike, Box 49, East Meadow, NY 11554 USA; 2 Nephrology Division, Department of Medicine, North Shore University Hospital, 100 Community Dr, Manhasset, NY 11030 USA; 3 Department of Business Management, Brooklyn College, 2900 Bedford Ave, Brooklyn, NY 11210 USA

**Keywords:** Chronic kidney disease, Medication dose adjustment, Dosing errors, Internal medicine, Internship and residency

## Abstract

**Background:**

Drug dosing errors result in adverse patient outcomes and are more common in patients with chronic kidney disease (CKD). As internists treat the majority of patients with CKD, we study if Internal Medicine house-staff have awareness and knowledge about the correct dosage of commonly used medications for those with CKD.

**Methods:**

A cross-sectional survey was performed and included 341 participants. The outcomes were the awareness of whether a medication needs dose adjustment in patients with CKD and whether there was knowledge for the level of glomerular filtration rate (GFR) a medication needs to be adjusted.

**Results:**

The overall pattern for all post-graduate year (PGY) groups in all medication classes was a lack of awareness and knowledge. For awareness, there were statistically significant increased mean differences for PGY2 and PGY3 as compared to PGY1 for allergy, endocrine, gastrointestinal, and rheumatologic medication classes but not for analgesic, cardiovascular, and neuropsychotropic medication classes. For knowledge, there were statistically significant increased mean differences for PGY2 and PGY3 as compared to PGY1 for allergy, cardiovascular, endocrine, and gastrointestinal, medication classes but not for analgesic, neuropsychotropic, and rheumatologic medication classes.

**Conclusions:**

Internal Medicine house-staff across all levels of training demonstrated poor awareness and knowledge for many medication classes in CKD patients. Internal Medicine house-staff should receive more nephrology exposure and formal didactic educational training during residency to better manage complex treatment regimens and prevent medication dosing errors.

## Background

Chronic kidney disease (CKD) is an important health problem with a rising incidence and prevalence in the general United States (US) population [1, 2]. Over the past two decades, there has been a 10–20% increase in incidence and prevalence of CKD stages 3–5 [3, 4]. CKD prevalence in the US general population is 13.6% [5]. This prevalence is even higher in patients with pre-diabetes (18%), diabetes (40–42%) and the elderly (23–58%) [6–10]. The elderly are a growing population with demographic models projecting their number to increase to about 1 in 5 people by the year 2030 [11].

The steep increase in the prevalence of CKD among the elderly might be partly due to related co-morbidities such as cardiovascular diseases, diabetes or hypertension [12]. Further, patients with CKD are at increased risk for cardiovascular and non-cardiovascular diseases, including infection and malignancy [13, 14]. With the increase in these co-morbid conditions with increasing age, elderly patients receive a large number of medications [15, 16]. These multiple drugs and complex regimens to achieve treatment goals for management of both CKD complications and various co-morbidities, put the elderly at a higher risk for drug related problems [15, 17].

Polypharmacy is highly prevalent and dosing error is one of the most important drug related problems in the elderly [18–23]. In a study of elderly patients in the US, over half reported the combined use of 5 or more prescription/non-prescription medications or dietary supplements [24]. In a similar study of elderly patients from Austria, the mean number of drugs taken was 7.5 with 58.4% of the elderly patients fulfilling their criteria for polypharmacy of more than 6 drugs [25]. Polypharmacy appears to be amplified in patients with CKD with one study reporting a mean of 8 medications in elderly patients with CKD stages 3 through 5 [26].

Many medications require dose adjustment in patients with CKD. Incorrect drug dosing was reported in 23.4% of patients, the majority of whom had renal impairment [25]. A review that assessed clinicians’ adherence to dosing guidelines for CKD patients reported nonadherence rates ranging from 19 to 67% [19]. A recent study further highlights this problem and reports that of the total 1464 antibiotic prescriptions filled for patients with CKD, 970 (66.3%) were for doses in excess of recommended guidelines [27]. Drug dosing errors can result in adverse effects, poor patient outcomes, and contribute to excess financial expenditures [28]. Adverse drug events are one of the top 7 leading causes of death in US and Canada [28–31]. In the US, annually more than 200,000 people die and another 2.2 million people are injured because of medication related problems [29, 32]. The cost associated with medication-related problems is estimated at over $2 billion per year in the US [33].

Due to a growing CKD population coupled with the often limited number of nephrologists and late referrals, the majority of CKD patients receive their ambulatory care from primary care physicians [16, 34]. In addition, patients with CKD are at increased risk for hospitalization where they are cared for by generalists [3, 35, 36]. It is important to determine if Internal Medicine (IM) house-staff are obtaining the knowledge necessary to correctly dose medications in a CKD population. To the best of our knowledge this has not been studied worldwide. We study awareness and knowledge among IM house-staff of dosage adjustment for commonly used medications across different medication classes in patients with CKD.

## Methods

### Participants and setting

We conducted a cross sectional survey to assess awareness and knowledge among 341 IM house-staff for dosage adjustment of commonly used medications across different medication classes in patients with CKD. The study was performed at six academic hospitals located in New York City and its suburbs. IM house-staff across all levels of training were surveyed in their fourth through sixth month of their training year.

### Variables

The following demographic information was collected: age (years), sex, medical school (graduate of American or International medical school), and level of training (post-graduate year (PGY)1, PGY2, PGY3 or greater). Personal/family history of kidney disease (personal history and/or history of kidney problems among spouse or children, and presence or absence of kidney problems in any first-degree relatives) was obtained. A renal training score (ranging from 0 to 3 for each affirmative response) consisted of the following: prior nephrology training or renal electives in medical school or residency, regular attendance at renal clinic (defined as 10 times or more during training), and interest in nephrology for further training.

A list of 26 commonly used medications was compiled representing 7 different medication classes: allergy (diphenhydramine, loratadine, montelukast), analgesic (acetaminophen, ibuprofen, meperedine, tramadol), cardiovascular (amlodipine, atenolol, carvedilol, digoxin, enalapril, hydralazine, simvastatin), endocrine (glipizide, pioglitazone, sitagliptin), gastrointestinal (famotidine, pantoprazole), neuropsychotropic (alprazolam, gabapentin, haloperidol, levetiracetam, paroxetine), and rheumatologic (allopurinol, colchicine). We offered the following possible responses to each medication: a) does not need dose adjustment, b) needs dose adjustment at glomerular filtration rate (GFR)<90 ml/min, c) needs dose adjustment at GFR<60 ml/min, d) needs dose adjustment at GFR<30 ml/min, and e) I don’t know.

### Outcome variables

One outcome was to assess the awareness among house-staff whether a given medication needed dose adjustment in patients with impaired renal function (‘medication dose needs adjustment’ awareness). The other outcome was to assess the knowledge among house-staff whether they knew at what level of GFR a given medication needs to be adjusted (‘medication dose adjustment at appropriate GFR level’ knowledge) according to published medication dosing guidelines in CKD [37, 38]. A score of ‘one’ was assigned for every correct response to a medication. A total score was calculated for each medication class and also for all medications (’overall medication score’).

### Statistical analyses

Descriptive statistics of either mean and standard deviation or frequency and percentage were used to describe the variables. The independent variable was post-graduate year (PGY). One outcome was correct ‘medication dose needs adjustment’ awareness and the other was correct ‘medication dose adjustment at appropriate GFR level’ knowledge. Analysis of variance (ANOVA) was conducted for total of all medications and also seven different medication classes. For the analyses with statistically significant differences, least significant difference (LSD) post-hoc analyses and analysis of covariance (ANCOVA) were conducted. The covariates included in the ANCOVA analyses were age, sex, medical school location, personal/family history of kidney disease, and renal training score. For the outcome variable with a skewed distribution, non-parametric analyses of the Kruskal Wallis test instead of ANOVA, Mann–Whitney tests instead of LSD post-hoc tests, and rank ANCOVA (Quade’s test) instead of ANCOVA were performed. A total score of all medications was calculated for all the 341 participants counting only the correct responses. Also, to allow for including the greatest number of participants in the ANCOVA analyses, the 34 individuals who omitted age were imputed with the mean sample age. SPSS Version 22 was used for all analyses. All *p*-values were two-sided.

## Results

Table 1 shows the descriptive statistics of the sample. Mean age was almost 30 years. Men were slightly more than women. Approximately equal numbers received their medical school training in the US and internationally. Almost half surveyed comprised of PGY1, with the PGY2 and PGY3 comprising approximately one-quarter for each group. Slightly more than one-tenth had a personal/family history of kidney disease. Mean renal training score was below 1 on a scale with a total possible score of 3.

**Table 1:** Descriptive Statistics of a Sample of 341 Internal Medicine Residents

Variables	Frequency	Percent	Mean	SD
Age (years)			29.2	2.95
Sex				
Women	158	46.3		
Men	182	53.4		
Missing	1	0.3		
School				
United States	165	48.4		
International	167	49.0		
Missing	9	2.6		
Training				
PGY1	158	46.3		
PGY2	101	29.6		
PGY3 and greater	82	24.0		
Kidney disease history (yes)	36	10.6		
Renal training score			0.7	0.76

Note: *PGY* post-graduate year, *SD* standard deviation

**Fig. 1:**
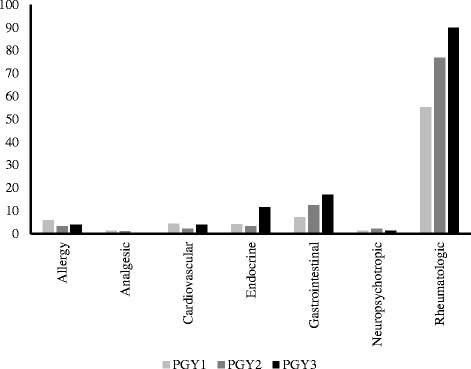
‘Medication dose needs adjustment’ awareness. Percent ‘completely correct’ responses by PGY groups

Table 2 shows comparisons for PGY groups for total scores for correct ‘medication dose needs adjustment’ awareness for different medication classes in CKD. ANOVA showed statistically significant mean differences for the PGY groups for total score of all medications, and also allergy, endocrine, gastrointestinal, and rheumatologic medication classes. These results were maintained in ANCOVA analyses adjusting for the relevant covariates. LSD post-hoc analyses showed that PGY2 had significantly greater mean scores than PGY1 for total score of all medications (*p* = 0.01), and also for allergy (*p* = 0.03), endocrine (*p* = 0.001), gastrointestinal (*p* = 0.004) and rheumatologic (*p* < 0.001) medication classes. LSD post-hoc analyses showed that PGY3 had significantly greater mean scores than PGY1for total score of all medications (*p* < 0.001), and also allergy (*p* = 0.001), endocrine (*p* < 0.001), gastrointestinal (*p* < 0.001), and rheumatologic (*p* < 0.001) medication classes. Also, LSD post-hoc analyses for endocrine showed that PGY3 had significantly greater mean scores than PGY2 (*p* = 0.01). There were no statistically significant mean differences for PGY groups for analgesic, cardiovascular, and neuropsychotropic medication classes. As can be seen by the mean scores, there was an overall pattern for all PGY groups in all medication classes except for rheumatologic where there was lack of correct knowledge. For rheumatologic medication class, PGY3 had 90.0% ‘completely correct’ (correct response for all the medications in a given class), PGY2 had 76.8% ‘completely correct’, and PGY1 had 55.3% ‘completely correct’ responses. An analysis of the 6 remaining medication classes showed that the highest percentage of ‘completely correct’ response for any particular medication class for any of the PGY groups was 17.1% for gastrointestinal (Fig. 1). For total score of all medications, no one scored 26/26 correct. Those who scored 20 or greater correct were 1 participant who scored 20/26 (0.3%) correct and 2 participants who scored 25/26 correct (0.6%).

**Table 2:** Comparisons for total scores of correct ‘medication dose needs adjustment’ awareness for different medication classes

Variable	Possible Total Score	PGY1 M (SD)	PGY2 M (SD)	PGY3 M (SD)	ANOVA *p*-value	ANCOVA *p*-value	Post-hoc
Allergy	3	1.3 (0.85)	1.6 (0.71)	1.7 (0.59)	0.003	0.046	PGY2>PGY1 PGY3>PGY1
Analgesic	4	1.3 (1.03)	1.1 (0.90)	1.4 (0.91)	0.19	–	–
Cardiovascular	7	3.4 (1.36)	3.6 (1.09)	3.6 (0.93)	0.27	–	–
Endocrine	3	1.1 (0.86)	1.5 (0.76)	1.8 (0.67)	<0.001	<0.001	PGY2>PGY1 PGY3>PGY1 PGY3>PGY2
Gastrointestinal	2	0.8 (0.56)	1.0 (0.52)	1.0 (0.54)	<0.001	0.001	PGY2>PGY1 PGY3>PGY1
Neuropsychotropic	5	2.1 (1.28)	2.2 (1.00)	2.2 (0.97)	0.79	–	–
Rheumatologic	2	1.4 (0.78)	1.7 (0.55)	1.9 (0.48)	<0.001	<0.001	PGY2>PGY1 PGY3>PGY1
Overall medication score	26	11.2 (4.31)	12.4 (3.28)	13.3 (2.10)	<0.001	0.002	PGY2>PGY1 PGY3>PGY1

Note: *M* mean, *SD* standard deviation, *PGY* post-graduate year, *ANOVA* analysis of variance, *ANCOVA* analysis of covariance. Sample sizes slightly vary due to omissions by participants

**Fig. 2:**
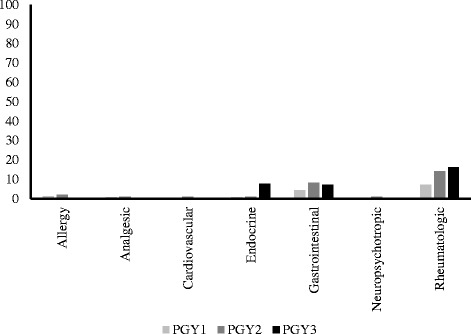
‘Medication dose adjustment at appropriate GFR level’ knowledge. Percent ‘completely correct’ responses by PGY groups

Table 3 shows comparisons for PGY groups for total scores for correct ‘medication dose adjustment at appropriate GFR level’ knowledge for different medication classes in CKD. ANOVA analyses showed statistically significant mean differences for the PGY groups for total score of all medications, and also allergy, cardiovascular, and gastrointestinal medication classes. The Kruskal Wallis test showed statistically significant mean differences for endocrine medication class. These results were maintained in ANCOVA analyses after adjusting for the relevant covariates. LSD post-hoc analyses (or Mann Whitney tests for endocrine) showed that PGY2 had significantly greater mean scores than PGY1 for total score of all medications (*p* < 0.001), and also for allergy (*p* = 0.001), cardiovascular (*p* = 0.01), endocrine (*p* = 0.02), and gastrointestinal (*p* = 0.003) medication classes. LSD post-hoc analyses showed that PGY3 had significantly greater mean scores than PGY1 for total score of all medications (*p* < 0.001), and also for allergy (*p* < 0.001), cardiovascular (*p* = 0.004), endocrine (*p* < 0.001), and gastrointestinal (*p* = 0.001) medication classes. There were no statistically significant mean differences for PGY groups for analgesic and neuropsychotropic medication classes, while rheumatologic medication class approached statistical significance for an overall statistical difference between the PGY groups. As can be seen by the mean scores, there was an overall pattern for all PGY groups in all medication classes where there was lack of correct knowledge. An analysis of the 7 different medication classes showed that the highest percentage of ‘completely correct’ response for any particular medication class for any of the PGY groups was 16.3% for rheumatologic medications (Fig. 2). For total score of all medications, no one scored 26/26 correct. Those who scored 20 or greater correct were 1 participant who scored 21/26 (0.3%) correct and 1 participant who scored 25/26 correct (0.3%).

**Table 3:** Comparisons for total scores of correct ‘medication dose adjustment at appropriate GFR level’ knowledge

Variable	Possible Total Score	PGY1 M (SD)	PGY2 M (SD)	PGY3 M (SD)	ANOVA or Kruskal Wallis *p*-value	ANCOVA *p*-value	Post-hoc
Allergy	3	1.2 (0.81)	1.5 (0.71)	1.6 (0.57)	<0.001	0.001	PGY2>PGY1 PGY3>PGY1
Analgesic	4	0.5 (0.69)	0.6 (0.77)	0.7 (0.66)	0.25	–	–
Cardiovascular	7	2.1 (1.06)	2.4 (0.99)	2.5 (0.84)	0.004	0.03	PGY2>PGY1 PGY3>PGY1
Endocrine	3	0.7 (0.76)	0.9 (0.79)	1.1 (0.87)	0.001	0.001	PGY2>PGY1 PGY3>PGY1
Gastrointestinal	2	0.7 (0.55)	0.9 (0.50)	0.9 (0.45)	0.001	0.002	PGY2 > PGY1 PGY3>PGY1
Neuropsychotropic	5	1.4 (0.95)	1.6 (0.95)	1.5 (0.89)	0.34	–	–
Rheumatologic	2	0.4 (0.62)	0.5 (0.73)	0.6 (0.76)	0.054	–	–
Overall medication score	26	6.8 (3.09)	8.3 (2.91)	8.8 (2.02)	<0.001	<0.001	PGY2>PGY1 PGY3>PGY1

Note: *M* mean, *SD* standard deviation, *PGY* post-graduate year, *ANOVA* analysis of variance, *ANCOVA* analysis of covariance. Sample sizes slightly vary due to omissions by participants. Endocrine, pain, and rheumatologic had skewed distributions and non-parametric analyses of the Kruskall Wallis test were performed instead of ANOVA. For endocrine, the Mann–Whitney test was performed instead of LSD post-hoc tests and rank ANCOVA (Quade’s test) was performed instead of ANCOVA

## Discussion

Our results showed that for analysis of ‘medication dose needs adjustment’ awareness there were statistically significant mean differences for total score of all medications, and also for allergy, endocrine, gastrointestinal and rheumatologic medication classes among the PGY groups with a consistent pattern of improved awareness for PGY2 than PGY1 and also for PGY3 than PGY1. Further, for endocrine medication class, there was statistically significant improved awareness for PGY3 than PGY2. There were no statistically significant mean differences among PGY groups for analgesic, cardiovascular and neuropsychotropic medication classes. For the analysis of the ‘medication dose adjustment at appropriate GFR level’ knowledge, there were statistically significant mean differences for total score of all medications, and also for allergy, cardiovascular, endocrine and gastrointestinal medication classes among the PGY groups with a consistent pattern of improved knowledge for PGY2 than PGY1 and also for PGY3 than PGY1. There were no statistically significant mean differences among PGY groups for analgesic, neuropsychotropic, and rheumatologic medication classes.

There was a consistent pattern of improvement in prescribing for allergy, endocrine and gastrointestinal medication classes and also for the ‘overall medication score’ with increased level of training. At the statistically significant level, both PGY2 and PGY3 demonstrated better awareness for ‘medication dose needs adjustment’ and better knowledge for ‘medication dose adjustment at appropriate GFR level’ as compared to PGY1. There were no statistical differences between PGY2 and PGY3 for these medication classes except for endocrine medication class for the ‘medication dose needs adjustment’ awareness. For ‘overall medication score’, allergy and gastrointestinal medication classes, our data trended towards a better PGY3 score compared to PGY2, but this was not statistically significant. Medical knowledge is expected to improve as residents advance through various levels of training. The new Accreditation Council for Graduate Medical Education (ACGME) accreditation tool of ‘milestones’ is based on a similar principle that focuses on continuous improvement in graduate medical education, including the competency of medical knowledge [39]. As maintained by educational milestones, residents are expected to demonstrate growth in their medical knowledge at established intervals as they progress through training [39]. Our results are similar in that increased level of training conferred greater knowledge from PGY1 to PGY2 and PGY3. However, even with this greater knowledge for both PGY2 and PGY3, there was still overall poor correct knowledge for these medication classes.

There was no significant improvement in prescribing pattern for analgesic and neuropsychotropic medication classes for both ‘medication dose needs adjustment’ awareness and ‘medication dose adjustment at appropriate GFR level’ knowledge. In addition, the overwhelming majority had incorrect knowledge across all levels of training. One possibility may be due to the limited exposure and infrequent prescribing of these medications by IM house-staff. Typically in academic settings, specialists (psychiatrists and neurologists) are involved with prescribing and initiating these medications. Another possibility is that some of these medications are controlled substances and may not be routinely prescribed by IM house-staff. This limited experience among IM house-staff for some of these medications may contribute to the overall poor scores for these medication classes. Outside of academic settings, generalists prescribe the majority (59 to 85%) of psychotropic medications in the US [40, 41]. However, studies report suboptimal dosing and duration with a lack of concordance between psychiatric diagnoses and prescribed psychotropic medications among generalists [42–44]. This has resulted in recommendations for graduate medical education programs to place greater emphasis on fundamental prescribing practices for psychotropic drugs [45–48]. Despite these recommendations, our results demonstrate poor knowledge about these medications among house-staff. With generalists responsible for the majority of these prescriptions, it is important that IM house-staff are aware of and adhere to dosing guidelines.

There was no statistical significance between the PGY levels for the ‘medication dose needs adjustment’ awareness for cardiovascular medication class, although the mean scores for PGY2 and PGY3 were greater than PGY1. For ‘medication dose adjustment at appropriate GFR level’ knowledge, PGY2 and PGY3 demonstrated statistically significant greater knowledge as compared to PGY1. Although PGY3 had a better score compared to PGY2, this was not statistically significant. However, even with this greater knowledge for PGY2 and PGY3 as compared to PGY1, mean scores indicated incorrect knowledge for more than half of the medications (i.e., at least 4.5 of 7 medications not correct). These results are surprising for the cardiovascular medication class. Cardiovascular disease is the second most common cause of hospitalizations in people over 65 years of age and is the leading cause of morbidity and mortality in the US accounting for almost 600,000 deaths in 2011 [49, 50]. IM house-staff receive vast exposure to cardiovascular disease through various cardiology rotations (including cardiac care unit, telemetry, cardiology consults, and cardiology clinics) in addition to treating numerous patients on medical floors and continuity clinics during residency. Furthermore, all IM house-staff are required to comply with’Centers for Medicare and Medicaid Services’ guidelines for inpatient (acute myocardial infarction and congestive heart failure) and outpatient (acute myocardial infarction and chest pain) cardiovascular core measures during all patient encounters [51]. One possible reason for poor knowledge may be due to the mild adverse effects associated with an extra dose of most anti-hypertensive/cardiovascular medications. In a study of young children with unintentional, single drug exposure to commonly used antihypertensive medications (metoprolol, bisoprolol, ramipril, enalapril, lisinopril, captopril, candesartan, valsartan, amlodipine, and verapamil), only mild symptoms occurred [52]. On the contrary, certain medications like digoxin can cause serious adverse events with a single extra/higher dose in patients with renal impairment. In our responses for digoxin, overwhelming majority (78.9%) of the house-staff were more cognizant that this medication needs to be adjusted in CKD.

There was a statistically significant mean difference between various PGY levels for the ‘medication dose needs adjustment’ awareness for rheumatologic medication class with PGY2 and PGY3 demonstrating better awareness as compared to PGY1. There were no statistical significant differences between PGY2 and PGY3, although PGY3 had higher mean scores. However, for the ‘medication dose adjustment at appropriate GFR level’ knowledge, we only found increased knowledge approaching significance among the PGY groups with mean scores improving as house-staff advanced through training. Interestingly, rheumatologic medication class had the highest percentage of correct responses as compared to all other medication classes. It is likely that the harmful side effect/adverse reaction profile of these medications contributes to better awareness and knowledge. A recent review of the safety and efficacy of allopurinol in CKD suggests that incidence of allopurinol hypersensitivity syndrome and other adverse effects like Stevens-Johnson syndrome can occur with a higher frequency in patients with impaired renal function [53]. Similarly, colchicine toxicity is more common among those with renal impairment and can be fatal [54–56].

Our sample data is fairly representative of the overall IM house-staff across the US. In the 2012–2013 academic year, there were 23,597 IM house-staff across all levels of training in the US [57]. The average age in our study was 29.2 years with 46.3% female house-staff. This is consistent with the US national average age of 29.4 years for IM house-staff with approximately 44% female house-staff [57]. The ACGME reports that there were 43.9% of international medical graduates (IMGs) in New York across all medical specialties as compared to 26.8% nationally [57]. Our sample had a similar percentage of IMGs as compared to the percentage of IMGs in New York of 49%. Our distribution of house-staff based on level of training is also consistent with the national US statistics: 46.3 versus 43.0% for PGY1, 29.6 versus 29.0% for PGY2, and 24.0 versus 28.0% for PGY3 (our sample versus US national average respectively) [57].

Our study has several limitations. First, the study was limited to the metropolitan area of New York. Although our sample was comparable in demographics to the total IM house-staff across the US, there may be a difference in patterns of training in different states. Second, the number of medications in each class was different. Third, the medications were selected based on our perception of what was being commonly prescribed. Fourth, we did not allow participants to use any automated support such as mobile- or computer-based devices for guidance in adjusting prescription dose. Although in clinical practice physicians have the opportunities to use these tools, our study is relevant as in clinical practice physicians are pressed for time and commonly do not utilize these tools [58], particularly if they are not even aware if a particular medication needs dose adjustment in CKD. Therefore it’s important to have understanding of resident physician awareness and knowledge.

## Conclusions

House-staff across all levels of training demonstrated poor awareness and knowledge of individualizing therapy based on patient’s renal function. Poor knowledge of renal dosing rules and lack of medication information have been identified as major causes of prescribing errors [59, 60]. Even with the use of electronic drug prescribing systems, these systems do not often provide guidance on the need for dose modification. It appears that current medical training has deficiencies in the area of renal dosing and thus potentially negatively impacts patient safety. With the shortage of nephrologists and the growing CKD population, it is essential that IM house-staff receive more nephrology clinical exposure and formal didactic educational training during residency to better manage the complex treatment regimens and minimize morbidity due to medication dosing errors. Future research should focus on approaches to improve awareness and knowledge of medication use in patients with CKD as adequate adjustment of the dosage is an absolute requirement for appropriate and safe prescribing.

## Abbreviations

ACGME: Accreditation Council for Graduate Medical Education
ANCOVA: Analysis of covariance
ANOVA: Analysis of variance
CKD: Chronic kidney disease
GFR: Glomerular filtration rate
IM: Internal Medicine
IMGs: International medical graduates
LSD: Least significant difference
PGY: Post-graduate year
US: United States
